# Retraction Note: *Melastoma malabathricum* Linn attenuates complete freund’s adjuvant-induced chronic inflammation in Wistar rats via inflammation response

**DOI:** 10.1186/s12906-022-03723-w

**Published:** 2022-09-20

**Authors:** Vikas Kumar, Prakash Chandra Bhatt, Kalicharan Sharma, Mahfoozur Rahman, Dinesh Kumar Patel, Nikunj Sethi, Atul Kumar, Nikhil Kumar Sachan, Gaurav Kaithwas, F. A. Al-abbasi, Firoz Anwar, Amita Verma

**Affiliations:** 1 Nautral Product Drug Discovery Laboratory, Department of Pharmaceutical Sciences, Faculty of Health Sciences, Sam Higginbottom Institute of Agriculture, Technology & Sciences (Deemed University), Allahabad, Uttar Pradesh 211007 India; 2 Centre for Advanced Research in Pharmaceutical Sciences, Microbial and Pharmaceutical Biotechnology Laboratory, Faculty of Pharmacy Jamia Hamdard, New Delhi, 110062 India; 3 University Institute of Pharmacy, Chhatrapati Shahu Ji Maharaj University, Kanpur, Uttar Pradesh 208024 India; 4 Department of Pharmaceutical Sciences, Dibrugarh University, Dibrugarh, Assam 786001 India; 5 Department of Pharmaceutical Sciences, Babasaheb Bhimrao Ambedkar University (Central University), Vidya Vihar, Rai Bareli Road, Lucknow, 226025 India; 6 Department of Biochemistry, Faculty of Science, King Abdulaziz University, Jeddah, Saudi Arabia; 7 Bio-organic & Medicinal Chemistry Research Laboratory, Department of Pharmaceutical Sciences, Faculty of Health Sciences, Sam Higginbottom Institute of Agriculture, technology & Sciences (Deemed University), Allahabad, Uttar Pradesh 211007 India

**Retraction Note: BMC Complement Altern Med 16, 510 (2016)**

**https://doi.org/10.1186/s12906-016-1470-9**

The Editor has retracted this article. After publication concerns were raised about some of the images shown in Figure 6. These concerns are that for both kidney and liver there appears to be overlap between the NC and MM panels. The authors have not provided the raw data. The Editor therefore no longer has confidence in the results and conclusions presented. The authors have not responded to correspondence from the Editor about this retraction.

